# Integrated Design Approaches for 3D Printed Tissue Scaffolds: Review and Outlook

**DOI:** 10.3390/ma12152355

**Published:** 2019-07-24

**Authors:** Paul F. Egan

**Affiliations:** Department of Mechanical Engineering, Texas Tech University, 2500 Broadway, Lubbock, TX 79409, USA; paul.egan@ttu.edu; Tel.: +1-806-742-3563

**Keywords:** design, integrated design, 3D printing, additive manufacturing, tissue scaffolds, bone, mechanobiology, mechanics, simulation, clinical applications

## Abstract

Emerging 3D printing technologies are enabling the fabrication of complex scaffold structures for diverse medical applications. 3D printing allows controlled material placement for configuring porous tissue scaffolds with tailored properties for desired mechanical stiffness, nutrient transport, and biological growth. However, tuning tissue scaffold functionality requires navigation of a complex design space with numerous trade-offs that require multidisciplinary assessment. Integrated design approaches that encourage iteration and consideration of diverse processes including design configuration, material selection, and simulation models provide a basis for improving design performance. In this review, recent advances in design, fabrication, and assessment of 3D printed tissue scaffolds are investigated with a focus on bone tissue engineering. Bone healing and fusion are examples that demonstrate the needs of integrated design approaches in leveraging new materials and 3D printing processes for specified clinical applications. Current challenges for integrated design are outlined and emphasize directions where new research may lead to significant improvements in personalized medicine and emerging areas in healthcare.

## 1. Introduction

Porous materials are necessary components in many regenerative medicine interventions [1]. For tissue healing applications, porous materials are used as scaffolding that provide mechanical support and promote onsite tissue growth [2]. In bone tissue engineering, scaffolds require a stiffness similar to bone and a porous geometry that facilitates high cell seeding efficiency, osteoblast adhesion, proliferation, and growth [3]. Effective scaffold design requires consideration of scaffold mechanics [4], nutrient transport [5], and biological growth [6]. Emerging 3D printing technologies are enabling the design and fabrication of complex geometries, such as metamaterial lattices [7], that have favorable trade-offs between stiffness and porous volume necessary for supporting growing tissue [8,9]. Developing high performing scaffolds using 3D printing is challenging [10], due to the numerous trade-offs in relevant properties [11], manufacturing influences on part performance [12], and experimental validation process [13]. Trial-and-error approaches are common in scaffold design [14], which is generally inefficient in comparison to methodological biological design approaches with greater integration of design, manufacturing, and modeling processes [15,16]. Recent advances in scaffold design have demonstrated promising directions for integrating design, modeling, and experiments to develop high-performance scaffolds for clinical applications [17,18]. This review focuses on highlighting advances and challenges in developing integrated design approaches for porous material design in 3D printed tissue scaffolds.

**Figure 1:**
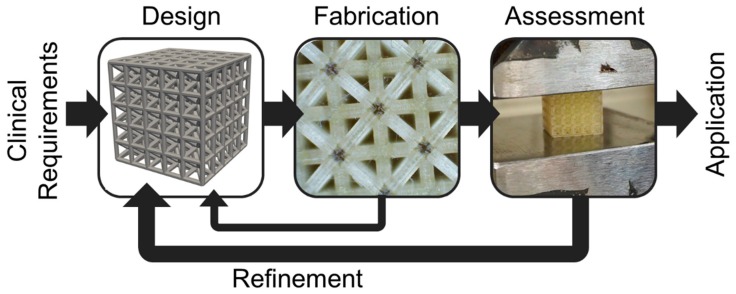
Integrated design approach with iteration for refining 3D printed tissue scaffolds.

Integrated design refers to the iterative and methodological use of multiple processes, such as modeling and experiments, to improve a targeted outcome [19], and has been explored for bone tissue engineering. The integrated approach is suitable for scaffolds as they can have diverse architected features beyond the development of their base material, such as pore gradients, local reinforcements, and multiple materials that necessitate integration of computational, experimental, and fabrication design processes within an overall iterative design approach [19]. For instance, iterative design and optimization of tissue scaffolds for vertebral fusion has included integration of diffusion models with local and global optimization of lattices [17]. Advanced modeling approaches have included computational fluid dynamics simulations for predicting biological tissue growth on complex geometries [20]. Lattice configuration with finite element approaches for mechanical assessment is another common integrative approach for tissue scaffold design [21]. Computational methods are particularly useful for 3D printed scaffolds, due to the need to create digital versions of designs prior to fabrication; computational methods also facilitate modeling and optimization [22]. However, it is essential computational methods are matched with experiments that validate their usefulness in predicting clinical outcomes to form a fully integrated approach [23]. Figure 1 demonstrates a recently developed approach for integrated design of tissue scaffolds [19], where design, fabrication, and assessment of scaffolds are combined in an iterative fashion to develop scaffolds for targeted clinical applications.

The integrated approach begins with a focused clinical application with relevant design requirements. A design is configured, fabricated, and assessed via simulations and/or experiments. Findings from fabrication, such as build accuracy, and from assessment, such as measured stiffness, inform future design decisions that form an iterative approach for improving scaffold designs for specific clinical applications. Iteration enables refinement of the design based on findings from fabrication and assessment that are not possible to accurately predict initially, and are incorporated to improve the design in subsequent iterations. Integrative approaches are necessary to streamline the overall scaffold lattice design process that must commence for each new combination of lattice and 3D printing process/material considered, since these factors have unique influences on lattice performance [24]. Recent use of the integrated approach for polyjet printing has demonstrated its feasibility for improving spinal fusion scaffolds by configuring lattice designs based on inaccuracies introduced from the 3D printing processes and their influences on scaffold mechanics [19].

An integrated design approach is beneficial for complex systems, especially those with both biological and mechanical functionality, and expands upon traditional design approaches such as structure, property, and function processes used to design specific materials [15]. Integrated design approaches must combine multiple design process, such as simulation with measurement systems, model validation and verification, or the handling of large data that can all improve upon traditional design approaches for materials [25]. Integrated design refers to the combination of processes rather than the products themselves, although it is possible to design an integrated product that has multiple features and parts using an integrated design process. Integrated design processes are well-suited for leveraging 3D printing with rapid iterations, and the recent integration of design thinking approaches with computer-aided design and 3D printing has led to improved product innovation [26]. Integrated design and fabrication approaches are also effective strategies for developing soft autonomous robots [27] and have led to platforms for designing biomechanically functional soft tissues when materials considerations were additionally included [28]. Although these results, and other studies have demonstrated the promise of integrated approaches for improving 3D printed designs, there are still many challenges to address before fully integrated design approaches are realized.

In this review, we will investigate current approaches in design, fabrication, and assessment of tissue scaffolds for clinical applications including bone fracture healing and fusion [29,30]. Assessment approaches are considered for both experimental and modeling results. The current status of integrated design for tissue scaffolds is assessed, followed by highlighted challenges that future research may address. Improvements in integrated design approaches have a great capacity for improving patient-specific design [31], medical big data [32], precision medicine [33], and new tissue engineering applications [34]. The field of tissue scaffold material design is far-reaching and includes contributions from numerous disciplines; this review focuses on a few of the areas with the greatest potential for bridging processes across fields that may promote innovations for integrated scaffold design.

## 2. Design

Designing scaffolds requires relating structural properties including pore size, porosity, and stiffness to mechanical and biological functions. Topology configuration for 3D printing scaffolds commonly includes patterning unit cells to form lattices with local and global optimization strategies. 

### 2.1. Topology

A first step in scaffold design is determining a strategy for configuring scaffold structural topology [35]. In the context of engineering design for optimization, topology refers to the optimized material layout of a structure within a given volume based on constraints, boundary conditions, and loading. For scaffolds, the configured topology must provide a network of pores that enable cell seeding and proliferation with sufficient nutrient flow. Common topologies include foams and lattices [8]. Bending-dominated foams tend to have lower stiffness per density than lattices constructed from beams to form truss-like structures that are stretch-dominated [9,24,36]. Advances in 3D printing enable the fabrication of complex beam-based lattices with tailored placement of beams for optimized performance. The higher effective elastic modulus of stretch-dominated structures allows for achieving a target scaffold stiffness at a lower density than otherwise possible, therefore providing a greater porous volume for nutrients and tissue growth.

**Figure 2:**
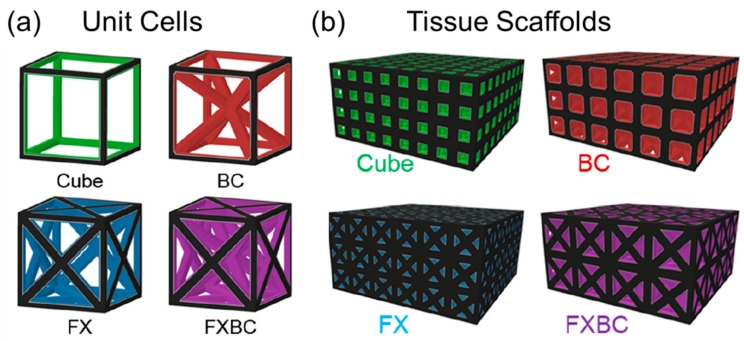
Design of (**a**) unit cell topologies (**b**) patterned to form scaffold lattices. Images adapted with permission [37].

The orientation of beams in a stretch-dominated structure will influence the mechanical response of the structure when loaded [11]. Beams aligned with the loading direction generally increase the structure’s effective elastic moduli, which means less deformation under load. Effective elastic modulus is a scalable structural property that refers to the behavior of an architected material. For lattice materials, the effective elastic modulus is based on its stress/strain response. The relative elastic modulus is found by dividing the effective elastic modulus by the elastic modulus of the base material used to construct the lattice. Therefore, the relative elastic modulus is always less than unity, and is dependent on the relative density (i.e., porosity) of the lattice. The addition of diagonal beams generally increases effective shear modulus. Different combinations of beam alignments forming unit cells provide a foundation for configuring tissue scaffolds with unique capabilities, as demonstrated in Figure 2 [37].

The simplest topology in Figure 2a is a cube unit cell with beams oriented orthogonally to each corner. The BC (“Body-centric”) unit cell is a cube with added beams from each unit cell corner towards the center of the structure. The FX (“Face-crossed”) unit cell has beams from each unit cell corner to the center of each of the unit cell’s faces. The FXBC structure includes both sets of additional beams. These cube-based unit cells were found to perform as well or better than alternate unit cell types when considering mechanical, nutritional, and biological factors [11]. When each unit cell topology is patterned to form scaffolds with controlled porosity and beam size in Figure 2b, the FXBC unit cells require overall larger unit cell sizes in comparison to cube scaffolds that are made up of many smaller unit cells. These differences in unit cells provide varied pore geometries and sizes throughout the scaffold that influence tissue growth rates [37,38], and merit further investigation to find favorable trade-offs among other scaffold properties that link to functionality.

### 2.2. Properties

Scaffolds are challenging to design due to the numerous properties linked to scaffold functioning and their respective trade-offs. It is common to configure scaffolds based on acceptable property ranges [39,40], since properties are typically simpler to determine than complex biological behaviors such as vascularization that more directly relate to scaffold functioning [41]. Experiments and simulations on complex biological phenomena inform feasible property values, such as scaffolds typically requiring at least 50% porosity for greater void volume for tissue growth. However, increasing porosity reduces scaffold mechanical functionality [9], which leads to complex trade-offs among relevant properties. Properties relevant for scaffold design include porosity, pore size, surface area, permeability, effective elastic modulus, and effective shear modulus [11].

Porosity refers to the ratio of void volume to total volume of a scaffold. A higher porosity provides more void volume for a tissue to fill and nutrients to flow, but results in a lower structural density with reduced mechanical properties. Pore size refers to the size of local void cavities in the scaffold. Larger pores provide greater volume for nutrient transport and vasculature growth while smaller pores provide more surface area and smaller volumes for faster tissue filling behavior. Fibrous tissue ingrowth increases with smaller pore sizes in porous bioceramics, which has been demonstrated with pores as small as 400 µm [42]. However, this smaller pore size significantly limited growth of blood vessels that benefit form larger pores and resulted in smaller blood vessel diameters. The optimal pore size for bone is typically considered as 200 µm to 1 mm. Pore size is defined in multiple ways depending on the research context. Pore size is often defined as the smallest sphere that can fit inside a cavity or the smallest planar void area a circle may fit [9]. Recent studies have defined the pore size of the square root of the planar void area, which controls for comparisons among differently shaped pores [3].

Surface area is typically calculated as surface area per volume [39], also referred to as specific surface area, and is linked to the number of cells that spread on the scaffold for initial seeding. For trabecular bone, specific surface area per volume is about 7.25 mm^−1^. Surface area and porosity are used to calculate permeability using the Kozeny-Carman relation [43]. A higher permeability means fluid more easily flows through the scaffold, and is required for nutrient distribution. The Kozeny-Carman relation is an empirically validated relationship that states the permeability of a porous structure is proportional to a constant based on the scaffold’s topology multiplied by the scaffold’s porosity cubed and divided by the scaffold’s specific surface area squared. The constant is found empirically or with computational fluid dynamics simulations. A greater porosity increases permeability due to there being less overall structure to obscure fluid flow. Increased surface area slows fluid flow due to the no-slip condition of fluid-structure interfaces. Permeability on the order of 1 × 10^−8^ m^2^ is typically acceptable for bone tissue scaffolds.

Commonly investigated mechanical properties include effective elastic and shear moduli that refer to the amount of deflection experienced by a lattice when loaded along an axis or via shear force [11], respectively. These are scalable material properties, meaning for a given unit cell design, a scaffold patterned with a small or large number of the same unit cells should retain the same effective elastic and shear moduli. These porosities are also theoretically retained if the unit cell size is rescaled while holding porosity constant, which is accomplished by proportionally resizing the beam diameter with the unit cell size. The recommended effective elastic and shear moduli for scaffolds is dependent on the overall scaffold size and supporting hardware such as pedicle screws and braces that also carry load, therefore influencing stiffness requirements for a scaffold such as a spinal cage system [44]. For instance, if a spinal cage system is redesigned with pedicle screws that carry a larger proportional load, the stiffness requirements for a scaffold used as part of a cage are reduced since the cage experiences lower overall loads.

### 2.3. Optimization

Design properties are typically coupled to one another such that improving one reduces another [11], therefore finding the optimal value of all parameters and properties is challenging. For instance, higher porosity results in greater permeability but lower effective elastic and shear moduli. Some properties may be altered independently of one another, such as pore size changing independent of porosity when resizing a beam-based lattice structure with a constant ratio of beam diameter to unit cell size. These differently sized pores would however alter the surface area of the structure. Design maps are one method that allows for determining how changes in multiple independent design parameters/properties influence a dependent property or function [9,12,37]. Computational design and optimization approaches are also frequently used to configure scaffolds with desired properties and performance for specific applications [22].

Computer-aided design aims to improve over trial-and-error approaches common in scaffold design by using optimization methods to efficiently search for favorable designs [14]. Search approaches begin with design generation followed by assessment based on properties or simulations for finite-element modeling, computational fluid dynamics, or mechanobiology evaluation [22]. Emerging 3D printing processes have opened the doors to many new topology design approaches since additive manufacturing enables the fabrication of otherwise difficult to construct structures.

Optimization has been used with mechanobiological assessment evaluation to investigate geometries with pore gradients, such that pores throughout a scaffold remain equal in size or gradually increase/decrease depending on their location [45]. The optimization approach used design parameters to describe the scaffold pore size distribution and evaluated designs using a mechanobiological model that predicted tissue growth based on mechanical and fluid stimuli. The search approach used a globally convergent gradient-based optimization algorithm, with gradients computed based on the finite difference approach [46]. Tri-linear variation in scaffold pore size significantly improved tissue growth in comparison to smaller distributions of pore sizes. Varied loading conditions and pore geometry also played a role in tissue formation, with shear loads improving tissue growth in addition to elongated pores. When the approach was used to investigate varied beam-based unit cell topologies, it demonstrated variances in preferred unit cell types and pore sizes based on loading conditions [47].

Topology optimization approaches are common for scaffold design and can incorporate multiple factors for design generation and assessment [48,49,50]. An integrated global-local optimization approach has been used to maximize the stiffness of a scaffold for animal implantation using finite element analysis [17]. The approach used a threshold density value of 50% for designing the scaffold and identified places of low and high preferred density for scaffold functioning. These areas were filled with locally optimized microstructures with favorable mechanical and mass transport properties [48]. The approach resulted in a designed cage fabricated using selective laser sintering that achieved a stiffness of about 7 kN/mm. Recent topology optimization has focused on ensuring even wall thickness to promote manufacturability in lattices [21], and have been validated experimentally and computationally. Optimization has also produced designs for reduced stress concentrations for bone tissue scaffolds that resulted in lattices with comparable properties to trabecular bone [49]. Hybrid lattices where one unit cell type transitions into another throughout a lattice have also been investigated with optimization approaches [50], and may be useful for developing scaffolds subject to loading from multiple directions.

## 3. Fabrication

Once a scaffold is designed there are diverse materials and 3D printing processes for fabricating the structure that influences final part performance. After fabrication, validation is necessary to determine the printed part’s accuracy in comparison to the intended design.

### 3.1. Material Selection

Base materials used for bone tissue scaffolds include ceramics, metals, and polymers [51,52]. Materials must not be toxic to cells and must provide a surface amenable to cell attachment and proliferation. Materials are often biodegradable, but in the case of some metals the scaffold is a permanent fixture [53]. Biodegradable materials must dissolve in the body at a rate that allows bone to grow and provide mechanical support to replace the scaffold. Degradation rate depends on material selection and how it is architected to form a lattice. A greater amount of proportional surface area leads to faster degradation. The most common ceramics used are tri-calcium phosphate and hydroxyapatite, polymers include polycaprolactone, polylactic acid, and methacrylic acid, while titanium is the most common metal for conventional scaffold manufacturing. Ceramics and polymers generally have closer elastic moduli to trabecular bone after adjustment for lattice porosity in comparison to titanium that is much higher. Recent advancements in magnesium scaffolds provide a better match to the elastic modulus of bone and are a biodegradable metal alternative to titanium [54]. However, magnesium could potentially cause issues with implants that degrade too fast and form gas cavities, which has been documented for several in vivo studies. Matching the stiffness of bone encourages tissue growth and avoids stress shielding that results in poorer bone growth when scaffold stiffness is much higher than surrounding bone.

3D printing processes for tissue scaffolds have been successful for ceramics [29], polymers [55], metals [56], and combinations of materials [2]. Suitable bone tissue scaffolds are constructed via 3D printing using all these materials to produce scaffolds with pores on the range of 200 µm to 1 mm and porosity of 50% or greater. One of the greatest challenges is achieving biocompatibility for scaffolds that require ultraviolet light curing, since photopolymers are generally toxic [57,58]. Ensuring that full curing commences followed by immersion in ethanol has resulted in improved biocompatibility of photocurable materials. Research recommends a three-tiered approach of using approved materials, appropriate manufacturing parameters, and post-processing techniques together for optimal performance. Ensuring biocompatibility will open new doors to using printing processes including stereolithography and polyjet printing [3,59], where preliminary cell culture compatibility has been demonstrated for complex lattices.

### 3.2. Printing Process

3D printing processes for fabricating lattices include extrusion, resin, and powder-based technologies. Each process provides different capabilities for printing features and resolution with access to varied materials. Extrusion-based processes operate by drawing material through a nozzle that may be heated to melt material that hardens once placed to form a structure. Fused deposition modeling is a common extrusion process for fabricating cellular lattice structures of varied topologies [60]. The process has been used to create lattices with orthogonal and diagonal beams with diameters of 1.5 mm using polylactic acid. Polylactic acid fused deposition modeling has been used to produce tissue scaffolds with channel diameters of 250 µm to 500 µm using an integrated design process that resulted in tissue growth [61]. Extrusion processes have also successfully produced scaffolds made of polycaprolactone mixed with hydroxyapatite [2] and alginate [62] and are capable of printing hydrogels. Extrusion processes are advantageous for their wide availability, ease of use, and wide choice of materials but are limited in terms of print resolution.

Resin curing processes include stereolithography, two-photon polymerization, and polyjet printing and operate by cross-linking liquid resins to form layered solid structures. Stereolithography is conducted using direct laser writing or digital light processing and has been used to prototype hierarchical scaffolds [44] and biocompatible lattices with micro-resolution [59]. Two-photon polymerization allows for the fabrication of structures with nanoscale resolution and has been applied in scaffolds with tunable stiffness for osteogenic growth [63]. Polyjet printing operates by curing liquid deposited on a surface that enables the placement of multiple materials and support materials throughout a constructed scaffold for complex geometries [3]. Overall, resin curing processes provide high resolution parts with complex features, but have limited material choices and often use toxic photopolymers for crosslinking materials during the curing process.

Powder-based processes utilize a bed of powder that is fused via laser, heat, or binders in a layer by layer fashion to create structures that are supported by unused powder during the printing process. Each powder-based process has differing capabilities and guidelines for correctly choosing a process that includes consideration of material processed, part complexity, and design features [64]. For instance, binder jetting enables the use of multiple materials in part construction, but leads to coarser parts and reduced detail in comparison to energy/laser powder printing. Laser sintering is often used to produce titanium scaffolds and is applicable for a variety of metals, including stainless steel [65]. Stainless steel lattices may be constructed with beams of approximately 200 µm that form orthogonal and diagonal beam intersections. For bone tissue engineering, powder-based processes provide multiple strategies for functional scaffolds and can operate with ceramics and metals while reaching suitable pore sizes and porosities [66]. Electron beam melting and direct metal laser sintering processes have supported osseointegration of implants in vivo [67]. Powder-based processes generally require higher investments than extrusion and resin-based processes, while providing comparable capabilities for scaffolds and enabling printing of a variety of metals suitable for high-strength lattices.

### 3.3. Accuracy

**Figure 3:**
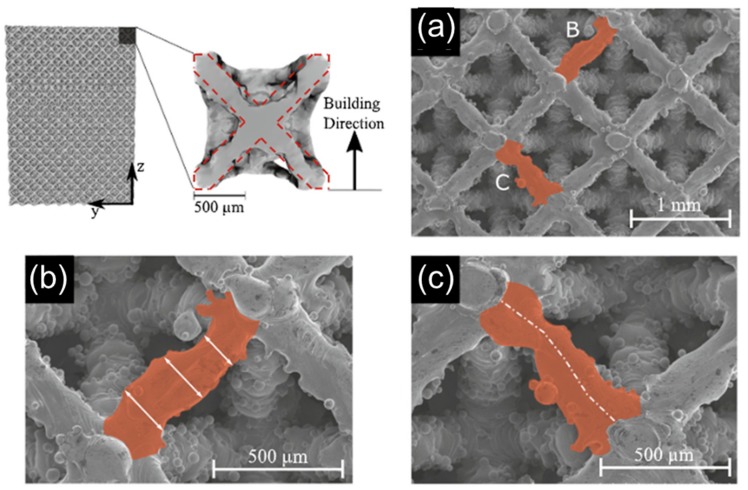
X-ray microtomography (MicroCT)-reconstructed octet lattice with (**a**) highlighted beams demonstrating (**b**) non-uniform cross-sectional area and (**c**) deviation from the center point. Images adapted with permission [12].

Printing processes have differing capabilities in achieved resolution based on materials used and their layer by layer build process [68]. At the microscale each build process introduces unique inconsistencies causing printed structures to differ from their intended design. For fused deposition modeling processes, inconsistencies in beam diameter are observed leading to stochastic changes in beam diameter width that influence lattice mechanical response [60]. When beam diameters were designed as 1.5 mm, measured diameters deviated following a Gaussian distribution from about 1.2 mm to 1.8 mm. Resin-based systems also demonstrate these inconsistencies, with polyjet printed parts having beam diameters on the order of 100 µm different from their intended design on average, with greater deviances observed locally [19]. These deviances led to overall porosity being sometimes 10% different than intended, depending on the design. Stereolithography printed parts tend to have better consistency in lattice microstructures than polyjet printed parts [59]. Variations in beam diameters are present in sintered designs using laser melting techniques [65]. These variations have been investigated (Figure 3) and demonstrated to influence structural mechanics for tissue scaffolds constructed from sintered titanium [49].

In Figure 3 an octet lattice was constructed and reconstructed using X-ray microtomography (MicroCT) scanning [49]. The ideal beam design is overlaid on a reconstructed unit cell and highlights deviances of the design both extending beyond and not filling the intended region of material placement. Scanning electron microscope images were used to highlight deviances in specific beams from Figure 3a. Figure 3b demonstrates that the measured width of a beam changes in magnitude along the length of the beam. Figure 3c demonstrates the midpoint of the beam along its length does not form a straight line, but rather deviates with a curvature based on material placement inconsistencies. Probability distributions of beam thickness were observed for both horizontal and diagonally constructed beams, with horizontal beams demonstrating greater inconsistencies in beam width and center deviation. These observations and measurements were used to inform mechanical models that better predict the behavior of the lattice based on build inconsistencies.

## 4. Assessment: Experimental

Experimental assessment is necessary to validate scaffold design performance, in addition to understanding mechanical response, nutrient transport, and tissue growth. In cases where experimental data is scarce, computational modeling informed by empirical observation aids in system understanding.

### 4.1. Mechanical Response

There is extensive research conducted for measuring the response of 3D printed lattices with varied topologies and materials. Experimentation of different configurations is necessary due to the vast design space enabled by 3D printing and because the material, printing process, and topology all influence the mechanical response [24]. Mechanical compressive tests are often used to study scaffolds since these tests have the greatest relevance to bone engineering applications. Finite element models are developed based on experiments to predict the behavior of novel lattice configurations.

Mechanical properties of 3D printed open-cell micro-architectures with selective laser sintering were studied to determine how mechanical irregularities introduced from the printing process influence mechanics [69]. Mechanical irregularities from variable beam diameters throughout a sample were demonstrated to influence mechanics by providing local weak links in the structure. Finite element models predicted the compressive behavior with greater accuracy than analytical models. The influences of manufacturing inconsistencies has also been demonstrated with fused deposition modeling [60] and polyjet printing processes [19]. In the latter study, the effective elastic modulus of a scaffold design scaled linearly with increases in beam diameter, which was hypothesized as partly influenced by inconstancies in microscale features on the order of hundreds of microns. The study also suggests that as long as porosity is constant, the number of unit cells patterned to form a scaffold does not significantly influence its mechanical properties [19]. However, such scaling is likely dependent on material selection and the printing process, therefore requiring reconsideration for new designs and materials.

Materials also influence mechanics as demonstrated by tricalcium-phosphate and titanium alloy under different loading conditions for dense (no pores) and open-porous scaffold types of 60% porosity and 600 µm pore size [70]. Tricalcium-phosphate scaffolds had approximately 1.14 GPa effective elastic modulus for dense configurations and 0.35 GPa for open-porous. In comparison, titanium samples had 114 GPa for dense scaffolds and 14 GPa for open-porous configurations. These findings demonstrate the expected proportionality of the scaffold effective elastic modulus to the elastic modulus of the base material. Additionally, for the same open-porous design there were different scalings of effective elastic moduli of the open-porous designs relative to the base materials. These open-porous tri-calcium phosphate scaffolds had about 30% of the relative elastic modulus compared to the dense sample of the same material while the titanium sample had about 10%.

Unit cells of varied topological design and dimensions have been mechanically tested when printed using titanium to determine their failure mechanisms [71]. Topologies included a cube, pyramid, and twist design that ranged from no to all beams being diagonal to the loading direction. The twist designs had the highest effective elastic modulus at a given porosity and for all designs effective elastic and shear modulus increased with porosity. Designs failed differently, the pyramid and twist designs showed shear deformation while the cube design demonstrated a layer-by-layer failure mechanism. These shear failure mechanisms have been observed in diverse 3D printed lattices [4], which also suggest for certain porosity ranges diverse topologies can achieve similar effective elastic moduli. Exponential curves are used to fit mechanical properties as a function of porosity [72], which was supported in studies that demonstrated shearing failure for bending dominated structures against layer-by-layer failures seen in cubic structures.

### 4.2. Nutrient Transport

Nutrients are transported through scaffolds via two primary mechanisms, fluid flow [43,73,74,75] and vascularization using the blood supply [76,77,78]. Although vascularization is the primary form of nutrient transport for in vivo scenarios, there is a need for fluid flow of nutrients in tissue engineered constructs seeded in vitro and in vivo prior to vasculature formation.

Fluid flow in scaffolds is influenced by porosity, permeability, and architecture [73]. The primary need for fluid flow is for nutrient delivery, waste removal, protein transport, and cell migration. Often, viable tissue formation is only observed in the peripheral regions of scaffolds since the interior of the scaffold lacks adequate diffusion. This phenomenon partly occurs as diffusion mechanisms are impeded during tissue growth as new tissue grows to fill pores and reduce fluid flow. In tissue scaffolds permeability is calculated using computational fluid dynamics for 3D printed tissue scaffold structures. Permeability of scaffolds that supported bone tissue growth were about 5 × 10^−9^ m^2^ to 30 × 10^−9^ m^2^ [43]. Computational analyses have confirmed the rates of oxygen diffusion in tissue engineering scaffolds [74], with both higher porosity and interconnectivity among pores improving diffusion. Empirical studies have demonstrated for scaffolds with minimized surface designs that porosity is the biggest differentiator in selecting suitable designs for targeted tissues [75]. Recent design approaches have generated hierarchical tissue scaffolds aimed to produce large internal pores that promote diffusion to the center of the scaffold and void volume for growing vasculature [44].

Empirical observation of vascularization requires costly in vivo studies that limit data availability for understanding how fast vasculature grows and branches in different types of scaffolds. Therefore, vascularization is often informed by direct observation followed by more in-depth investigations with computational studies. Vascularization has been empirically studied by concentrating on the angiogenic process of vessel formation that initiates during the healing response after a scaffold is implanted. A mechanobiological model demonstrated that a higher initial number of cells seeded homogenously on a scaffold reduces the vascularization rate, and therefore the maximum penetration of the blood vessel network and resulting bone growth [76]. Three-dimensional modeling of angiogenesis in porous scaffolds with diverse porosities and pore distributions suggest a higher porosity, larger pore size, and greater interconnectivity among pores all contribute to rapid and extensive angiogenesis [77]. These findings inform strategies for the development of 3D bioprinting approaches that can produce vessel like structures containing multi-level fluidic channels with optimal distributions [78]. New developments of scaffolds with vasculature formed prior to implantation through these mechanisms could significantly improve nutrient distribution and subsequent tissue growth and regeneration.

### 4.3. Tissue Growth

Tissue growth experiments are conducted in vitro in a laboratory environment [6,79,80,81,82,83] and in vivo using animal models [13,84,85,86,87]. In vitro studies often investigate specific phenomena in controlled conditions or early experimental feasibility prior to more intensive in vivo experiments that better recreate the biological niche of clinical applications. In vitro studies have a lower associated expense and provide greater volumes of data that enable targeted understanding of phenomena, such as isolating the mechanisms of three-dimensional tissue growth.

Three-dimensional environments are necessary for investigating bone growth, where osteoblasts are seeded on scaffolds, proliferate, and grow to form tissues capable of depositing minerals for bone construction. In vitro studies of scaffolds with cubic pores have demonstrated that osteoblasts grow on hydroxyapatite (HA) scaffolds to form curved tissue surfaces with circular shaped pore cross-sections [79]. These HA scaffolds had approximately 50% porosity, 500 µm beam widths, and 500 µm pores. Osteoblasts adhered to the HA surface two days after seeding and with time completely covered the HA surface. Cells tended to fill micropores and cracks present from the HA sintering process. The formed tissue had a few layers of cells on flat surfaces and also areas where many layers of cells that grow towards the center of a pore from corners on the scaffold. Over a period of four weeks tissue grew to fill a majority of the void porous space in the scaffold.

**Figure 4:**
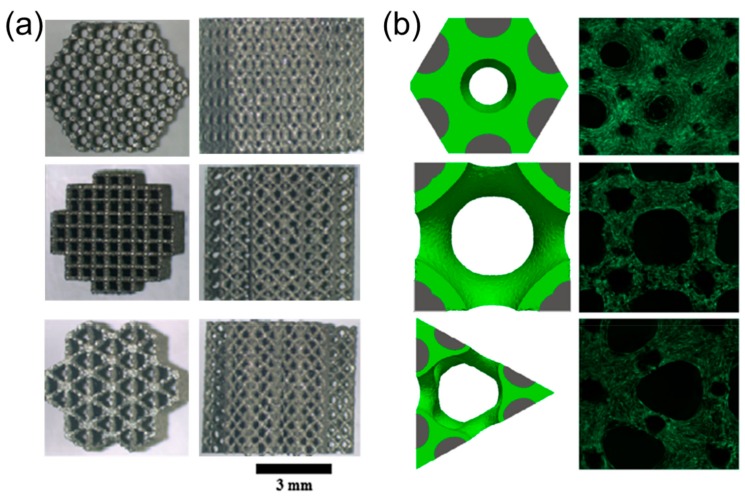
Scaffolds with varied pore geometries (**a**) fabricated with titanium. (**b**) Tissue growth simulation and in vitro growth after 14 days. Images adapted with permission [83].

This observed curvature-driven behavior (i.e., growth from scaffold corners to form circular fronts) has been extensively studied and recreated by a number of studies in vitro that investigated square/triangle pores for osteoblasts [80]. Studies have also demonstrated relevance for fibronectin organization [6] and bone marrow [82] with detailed imaging of the cytoskeletal formation. A recent study investigated three-dimensional growth in titanium scaffolds with hexagon, square, and triangular planar pores [83], with highlighted results in Figure 4.

The scaffolds in Figure 4a were designed with pore sizes of approximately 500 µm measured in the horizontal plane while the vertical planes of all scaffolds had similar cross-beam designs. Scaffolds were seeded with osteoblasts and after 14 days growth occurred fastest in hexagon shaped pores (~80% planar fill), followed by square pores (~25% planar fill), and lastly triangle pores (~20% planar fill) as shown in Figure 4b. These differences remained for scaffolds designed with 1 mm pores with the hexagon filling fastest (~85% planar fill), and the square (~55% planar fill) filling much faster than the triangle (~10% planar fill). This behavior was recreatedin silico using simulations shown in Figure 4b that modeled growth rates based on the local curvature of the scaffold and growing tissue front.

Recent in vivo studies have demonstrated bone growth for titanium scaffolds of 300 µm to 900 µm in rabbits [84]. They concluded a scaffold with 600 µm pores had the greatest balance and success among those studied. The 600 µm pore scaffold provided higher fixation ability after two weeks than other implants and at four weeks had sufficiently high fixation in a detaching test. Fixation ability refers to how well each implant integrates with surrounding bone. In vivo studies using polycaprolactone and tricalcium phosphate scaffolds in bovine models have demonstrated curvature-based tissue growth that leads to bone mineralization [13]. Recent studies have also extensively investigated varied lattice topologies and materials for fracture healing in rabbits [85,86,87] that provide evidence of scaffold capabilities for clinical applications.

## 5. Assessment: Modeling

Modeling approaches are used to predict scaffold performance and reduce the need for costly experiments. Analytical and simulation approaches are common, with simulations being necessary when investigating complex phenomena that are difficult to model mathematically.

### 5.1. Mechanical Behavior

Analytical and numerical approaches, such as finite element analysis, are used to model the mechanical behavior of lattice structures under load. Analytical approaches have compared foams and lattices on the basis of their properties [8], with the primary distinction being that lattices are connected networks of beams. In this sense, lattices are cellular materials with properties that extend from their base material, such as stiffness, strength, thermal conductivity, and diffusivity. The three factors that dominate a lattice’s properties are the material used to construct the lattice, the lattice’s cell topology and shape, and the lattice’s relative density compared to a solid of the same material. The most important distinction in lattices is between those with stretch or bending-dominated mechanical behavior. Stretch-dominated lattices have a higher scaling of strength with density. For a material architected with 10% relative density, a stretch-dominated structure is about three times stronger than a bending-dominated structure of the same relative density [9]. The stretch-dominated behavior occurs in lattices that are configured such that bending of struts is prevented and strut-stretching is the dominant deformation mode.

Finite element methods are commonly used to model the behavior of cellular materials [88]. Finite element analysis is a numerical approach that approximates the behavior of a large system by subdividing it into smaller parts referred to as finite elements. Simple equations are used to model these finite elements that are assembled into a larger system of equations to describe the aggregate structural behavior. Comparisons have been made of lattices modeled using two approaches, with 3D beam element models and as continuum element models. When comparing these approaches, accuracy depends on the structures’ geometries and governing deformation method. The beam element model over predicts stiffness in principal directions because of the rigid domains modeled straight through beams in these directions. Finite element modeling has also been used to investigate the crushing behavior of lattice structures [65], and suggests that reducing the unit cell aspect ratio by making it taller and narrower can improve initial stiffness, yield strength, and energy absorption for compressive loads. Recent studies using finite element methods have investigated mechanical metamaterials and demonstrated their feasibility as bone mimicking materials with functional grading compositions with dense outer layers to mimic cortical bone and porous interior layers to mimic trabecular bone [89].

Finite element and analytical approaches have also been used to investigate lattices in the context of tissue scaffolds, with integration of biological behaviors [14,90]. Lattice design for scaffolds is constrained by the need to retain porosity and pore sizes beneficial for tissue growth. Recent scaffold design approaches have used beam-based finite element analysis to investigate large numbers of alternate hierarchical scaffold designs [44]. The introduction of hierarchy was found to lower the stiffness while increasing porosity. Later empirical studies demonstrated the beam-based finite element analysis over estimated stiffness for these structures [19], and other recent findings suggest solid modeling techniques are more appropriate for modeling lattices configured as tissue scaffolds [12]. These studies found that inconsistencies in the printing process at the microscale contributed to deviations between modeling and experiment, therefore motivating the need for new methodologies that take the 3D printing process and base material into account.

### 5.2. Scaffold Nutrition

Nutrients are transported through scaffolds via fluid flow initially. After tissue growth and angiogenesis occurs, fluid flow is impeded and nutrients are further distributed by diffusion from blood vessels. Fluid flow models typically use computational fluid dynamics to predict fluid behavior [43,91], while nutrient distribution through blood vessels requires simulating vasculature growth followed by nutrient diffusion [41,76]. Fluid flow also plays a role in predicting the shear stress that stimulates tissue growth [20,92,93]. Computational fluid dynamics simulations and numerical methods have been conducted for bone and provide insights for fluid flow behaviors that scaffolds should mimic [91]. Permeability is directly related to fluid velocity and pressure, that is dictated by the geometry and porosity of its porous media and is on the order of 1 × 10^−8^ m^2^ for bone. However, bone is anisotropic so permeability differs based on fluid flow directionality through the material.

Permeability for scaffolds has been found using computational fluid dynamics simulations for 3D printed scaffolds with diverse geometries. The Kozeny-Carman relation is a means of predicting permeability of a porous material based on its porosity cubed over its specific surface area (surface-volume ratio) squared all multiplied by a constant [11]. The constant is found empirically or by using computational fluid dynamics simulations. Simulations for varied lattice topologies have demonstrated the permeability to be similar for all beam-based configurations. Scaffolds were then generated with permeabilities on the same order of magnitude as bone with porosities ranged that from 50% to 70% and pore sizes that ranged from 200 µm to 1 mm. Optimal values of permeability for each scaffold require further consideration of how fast and how much tissue should grow. Computational fluid dynamics has also been used to calculate permeability in 3D printed titanium beam-based lattices that supported in vitro osteoblast growth [43], thus demonstrating permeabilities similar to bone are suitable design targets.

Vasculature models have been combined with mechano-regulation algorithms [76] and also used on their own to predict rule-based formation of blood vessels in tissue scaffolds [77]. The rule-based approach is informed by in vivo experiments that observed the branching and formation of blood vessels. In the rule-based approach software agents represent endothelial cells and interact together with their micro-environment that leads to the invasion of blood vessels into the scaffold. The framework allows for investigating homogeneous and heterogeneous scaffold lattices, and has informed the desirable range of scaffold properties suitable for tissue engineering applications [41]. These models provide a numerical evaluation of total blood vessel length, invasion depth, and total number of sprouts formed during angiogenesis that can later inform diffusion of oxygen and nutrients to different locations in the scaffold.

### 5.3. Biological Growth

Biological tissue growth is modeled using analytical approaches [90,94], rule-based simulations [95,96,97], mechanobiological models [47,98], and curvature-based simulations [83,99,100,101]. Simulations are often used for 3D printed designs due to the complexity of their topologies, with mechanobiological models and curvature-based simulations being the most common since they consider aggregate tissue growth behavior rather than individual cell behaviors. Mechanobiological models for bone regulation predict that tissue growth occurs based on mechanical loading and shear strain from fluid flow on the tissue [98]. A finite element model was used to determine mechano-regulation of stem cells with regards to tissue differentiation and growth. Simulations suggested that high porosities (70%), higher stiffness (1000 MPa), and medium dissolution rates (0.5%/iteration) provided the greatest amount of bone growth. Recent use of the mechanobiological model has demonstrated tissue growing to form curved surfaces and has suggested optimal beam diameters for varied lattice architectures to promote tissue growth [47].

**Figure 5:**
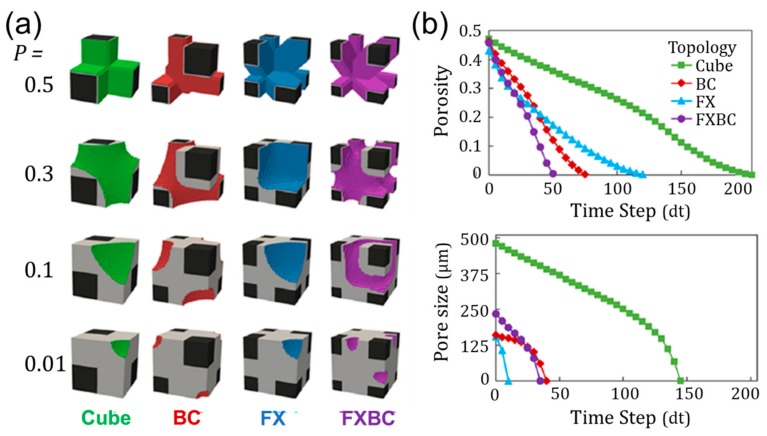
Tissue growth simulations (**a**) for unit cells with initial porosity P = 0.5 with plotted (**b**) changes in porosity and pore size for each time step. Images adapted with permission [37].

Curvature-based simulations predict tissue growth based on the local scaffold and tissue curvature by assuming tissues are built of tensile elements representing contractile cells. For planar pores, pore shapes are classifiable using “non-convexity” and “circularity”, which influences how many pores can fit in a scaffold geometry. For the planar pores investigated, overall growth time occurs at similar rates based on surface area regardless of pore geometry [99]. The model was extended into three dimensions to determine tissue growth in differently shaped and sized pores using a voxel-based method [100], and also validated in vivo [13]. It was observed that tissue growth halts and does not continue to complete pore filling behavior if the initial pore size is too large and positive curvature is not maintained on the growing tissue front. Level set methods were developed as an efficient means for modeling curvature-based growth for varied 3D printed geometries [83], and demonstrated three-dimensional pore filling behavior occurs at different rates based on pore geometry that was validated in vitro. Recently curvature-based voxel simulations have investigated growth rates on diverse 3D printed lattice designs constructed from beams, with results shown in Figure 5 [37].

Figure 5a topologies are the same beam-based topologies described in Figure 2. The simulation uses a virtual environment with voxels representing structure, tissue, void space, and the advancing tissue front. One corner of each unit cell is generated with specified beam width and unit cell size with structure voxels to initiate a simulation. Only an eighth of each unit cell is required for simulations based on symmetry and boundary conditions. Tissue voxels are placed to seed structure surfaces. The interface between tissue and void volume is tracked and tissue is placed each time step to simulate growth based on local curvature calculations. Tissue advances initially from intersections of beams and leads to different growth patterns and rates based on local topology. When controlled for porosity, growth occurs fastest in structures with more beams per volume, as demonstrated for both volume filling and pore filling behaviors (Figure 5b). The study also investigated growth in diverse designs of varied beam widths and unit cell lengths (and therefore porosities/pore sizes). Similar trade-offs between tissue growth rates and permeability emerged for all topologies when balanced designs were identified. Tissue growth also occurs at slightly slower rates if beams have rounded cross-sectional areas or if pores are initially rounded [102]. This slower growth occurs because corners have high localized curvature for accelerated tissue growth. These findings further support the used of beam-based 3D printed geometries for beneficial tissue growth functionality, in addition to their mechanical advantages.

## 6. Clinical Applications

Tissue scaffold success ultimately depends on clinical performance [103,104]. Bone fracture healing and bone fusion are common applications that demonstrate the current state of the art in scaffold design and the need for further integration of research and development for 3D printed porous material designs.

### 6.1. Fracture Healing

Recent studies have investigated 3D printed scaffold capabilities for healing fractures in animal models, which includes growing bone or cartilage to produce tissue for osteochondral defects that occur in a variety of situations that include trauma, disease, and aging. Hydrogels that have inherently weak mechanical strength have been improved with thermoresponsive supramolecular copolymers synthesized with dual hydrogen monomers and further components [105]. These improved hydrogels demonstrated robust tensile strength to 0.41 MPa, stretchability up to 860%, and high compressive strength up to 8.4 MPa. In vivo experiments using a rat model demonstrated significantly improved regeneration of cartilage and subchondral bone using these hydrogels. Mixes of polycaprolactone, hydrogels, stem cells, and pluronic F127 have been used as scaffolds to begin repairing mandible defects and were evaluated favorably after demonstrating osteogenic differentiation for 28 days [106,107]. 3D scanning followed by 3D printing has been demonstrated as an effective strategy to repair bone defects in situ for a variety of conditions including large segmental defects of long bones, free-form fracture of femoral condyle, and chondral lesions [108].

**Figure 6:**
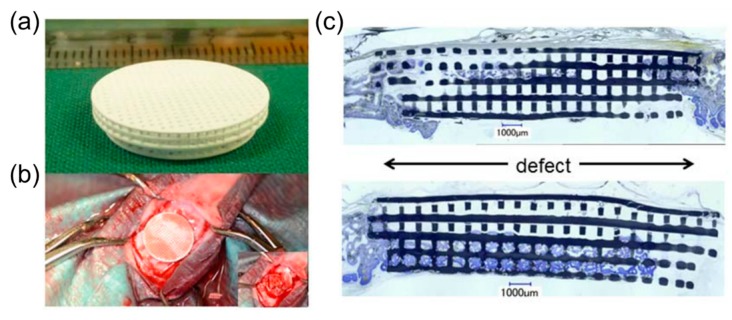
Critical size bone defect healing assessed with (**a**) a tri-calcium phosphate scaffold (**b**) implanted in a rabbit bone with (**c**) post-op histological section after sixteen weeks. The scaffold appears greyish black and the bone appears purple in the histology image; the distorted lattice structure and gaps indicate failure. Images adapted with permission [29].

Another common factor in developing materials for defect healing is the need to match the properties of bone to encourage regenerative growth. 3D printed lattice materials have been developed with compressive strength comparable to cortical bone (100–150 MPa). The materials were glass-ceramic scaffolds using a hexagonal design that achieved a 1,000,000 cycle fatigue resistance for a 1–10 MPA compressive cyclic load and five times greater strength than reported for ceramic and glass scaffolds of similar porosity [109]. Beam-based lattices have been demonstrated to mimic bone and support pre-osteoblastic cell proliferation using an acrylate polymer certified as a Class VI material by the United States Pharmacopeia [110]. These findings suggest internal architecture can play a large role in achieved lattice properties suitable for mimicking bone, which is tunable with computational methods such as interpolation for microstructures [111]. In vivo testing has been conducted to determine fracture healing in rabbits for varied 3D printed topologies [85,86,87], and has suggested there are a large number of potential internal architectures that achieve similar clinical outcomes for successful bone growth. Figure 6 demonstrates the findings for bone defect healing in a rabbit using a tri-calcium phosphate scaffold [29].

In Figure 6a the tri-calcium phosphate scaffold is constructed with orthogonal unit cells with 300 µm diameters and 500 µm channels for healing fracture defects that were critically sized with 15 mm diameters as demonstrated in Figure 6b [29]. Results in Figure 6c demonstrated that the scaffold promoted new bone growth, but also distorted and failed. Defect bridging improved significantly for the tri-calcium phosphate scaffold with ~67% bridging compared to ~40% bridging for empty defects after 16 weeks. Titanium scaffolds outperformed tri-calcium phosphate scaffolds and significantly improved both bone area and defect bridging during healing, thus demonstrating the influence of material selection on success, in addition to internal architectures. Specifically, titanium scaffolds of the same design had ~92% bridging after 16 weeks and had ~43% bony regeneration in a middle area of reference in comparison to bony regeneration of ~32% for tri-calcium phosphate scaffolds and ~24% for empty defects.

### 6.2. Bone Fusion

Vertebral fusion is a common bone tissue engineering procedure conducted to manage degenerated disk disease. In the procedure, a spinal interbody cage is implanted in the body to replace a partial or completely removed intravertebral disc. The cage then facilitates a biomechanical response that leads to bone growth between two vertebral bodies to enhance spinal stability [112]. Titanium foams that mimic the structure of bone are a conventional approach for successful fusion and can achieve permeabilities similar to trabecular bone for porosities of 50% to 80% [113]. Mechanobiological models have informed cage geometry design and have been used to evaluate cages with constant compressive stiffness and cages designed to optimally provide the ideal mechanical stimulus for bone formation throughout the entire fusion process [113]. Cage geometry was found to significantly influence outcomes in addition to varied loads of 250 N, 500 N, and 1000 N. Interbody cages have also been compared on the basis of their mechanical responses for 50% porous structures [112]. Investigations have suggested that using a strategy with two smaller cages, rather than one large cage, can provide higher structural stability.

3D printed cage designs have been investigated computationally and experimentally. An approach using global-local topology optimization resulted in cages with variable pore sizes with acceptable stiffness ranging from 4 kN/mm to 7.1 kN/mm using polymer materials [17]. Beam-based cages have been optimized for printing with titanium by altering the internal architecture and using topology optimization on the cage to bone interface with overall stiffness reaching about 25 kN [114]. Cages have also been designed and fabricated with polyjet printing with large hierarchical pores in the center to account for nutrient growth, while also achieving a stiffness of about 10 kN/mm that is comparable to other topology optimized cages constructed with polymers [19]. These cages were also demonstrated to support the preliminary stages of in vitro bone growth [3].

In vivo testing has also supported spinal cage design decisions and validation. Biodegradable and absorbable poly-l-lactic acid cages have been constructed with exterior dimensions of 10 mm by 10 mm by 18 mm [115]. These cages had no interior structure and maintained their original structure and mechanical properties at six months after implantation, but had significantly disintegrated by twelve months. Comparisons of these cage designs with flexible poly-l-lactic acid, stiff poly-l-lactic acid, and titanium demonstrated an influence of stiffness on fusion rates [116]. The reduced stiffness of the poly-l-lactic acid cages demonstrated enhanced interbody fusion compared to the titanium cages at six months. A tri-calcium phosphate cage, autologous bone graft, and polyether ether ketone cage were compared using an in vivo goat model and demonstrated that both cages outperformed the bone graft, with the tri-calcium phosphate device providing the greatest increase in intervertebral bone volume [117]. 3D printed titanium cages have also been tested in vivo and demonstrated good biocompatibility and osseointegration with similar performance to polyether ether ketone materials [30], thus demonstrating the potential in using 3D printing to reach new capabilities in spinal fusion treatments.

## 7. Outlook

Although recent advances have improved the design and fabrication of tissue scaffolds, numerous challenges impede the realization of fully integrated design approaches for scaffold optimization. Here, current challenges are addressed related to developing integrated design approaches and achieving specified clinical outcomes.

### 7.1. Integrated Design

Recent advancements in design, fabrication, and assessment of tissue scaffolds provide foundations for improving porous scaffold materials, and further integration could lead to greater advancements in tissue scaffolds for clinical applications. Integrative approaches enable iteration among process, such that experimental findings and influences of manufacturing processes inform computational design methodologies for patient-specific applications [19]. Integrated approaches are timely with the emergence of 3D printing technologies that are becoming more cost-effective for the clinic, therefore motivating design methods that rapidly adapt to new technologies and patient needs.

Current integrative approaches include multidisciplinary modeling assessment and using modeling results to inform design decisions [20,47,93]. These approaches are limited by computationally intensive evaluation times necessary for modeling behavior, therefore integration with design search methods could aid in reducing the number of model evaluations required to find optimally performing designs. Design approaches have also adapted to influences of print processes and accuracy on performance [9,19]. These approaches benefit from streamlined integration of design and experiment since print processes, materials, and topologies have unique influences on part performance and require recharacterization and validation as new configurations are considered. Design and experiment have been integrated to optimize pore shapes and also spinal cages for clinical applications based on in vivo findings [30,99]. Greater synergy among processes provides greater flexibility and justification in design decisions [56,61], and further integration is a necessary step in streamlining design approaches for the future.

Future scaffold design approaches will need to adapt for each patient’s unique physiology [31]. Integration is necessary as the entire design process must rapidly produce high performing solutions with unique geometries and loading conditions. Big data plays a role in design personalization when considering each beam in a lattice scaffold material could have a unique length and diameter and must interface with local tissue geometry [32]. Personalization also plays a role at the materials level when developing bioscaffolds with unique features that fit into patient-specific biological niches for precision medicine [33]. Integration will enable extensions of generalizable design approaches, so that processes for designing bone tissue scaffolds may extend to other applications, such as spinal injury repair [34]. Flexible integrative approaches also provide leverage in using new printing process, such as nanoscale tunable scaffolds [118], where new mechanical and physiological phenomena require consideration for improved healthcare solutions.

### 7.2. Challenges

**Figure 7:**
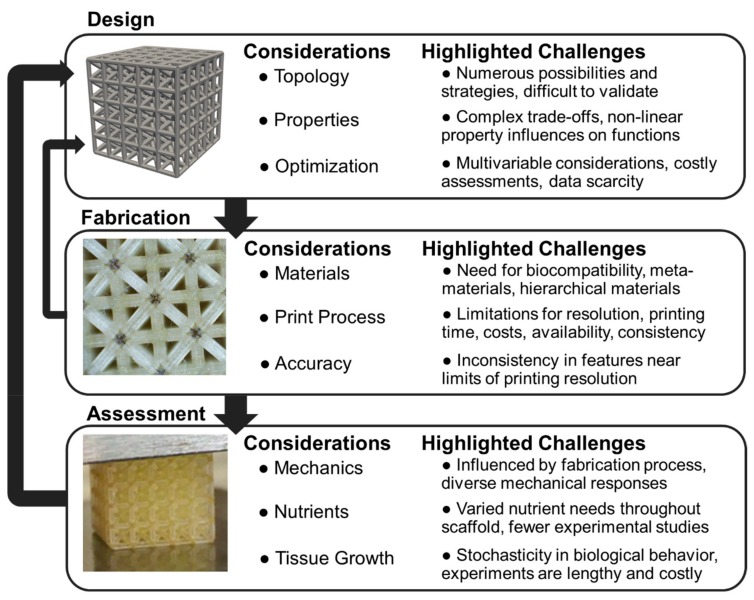
Summarized considerations and challenges for integrated design, fabrication, and assessment of tissue scaffolds for clinical applications.

The review has summarized a subset of recent research advancing tissue scaffold design that informs the future of fully integrated approaches for higher performance and patient-specific designs. In Figure 7, considerations for integrated design, fabrication, and assessment areas are highlighted with unique challenges for researchers to address across disciplines.

Design focuses on configuring scaffold structures with specified topological organization and properties for optimized tissue growth. Topology design has many possibilities for choosing unit cells or configuration strategies that are difficult to compare due to the number of design decisions available for fine-tuning each topology. The design of hierarchical lattices provides additional challenges, since greater complexity emerges from features at multiple levels [44]. Property assessment requires computational advancements for exploring complex design trade-offs. There is a need for assessing how scaffold structural properties such as porosity and interconnectivity link to more complex scaffold functionality such as vascularization and nutrient distribution that are currently explored with simulations [41]. There is a need to build on existing optimization approaches by incorporating more relevant phenomena [17], such as assessment of mechanics, angiogenesis, and tissue growth simultaneously while taking care to ensure efficient search strategies. Optimization advances will also need to incorporate big data approaches for patient-specific tuning.

Fabrication processes focus on the selection of base materials and strategies for developing lattices as metamaterials with unique properties by leveraging emerging technologies. With regards to material selection, there is a lack of biocompatible materials across the full range of traditional material properties. For instance, photopolymers have varied toxicity levels influencing in vivo implantation and limiting the feasibility of tissue scaffolds from 3D printed polymer materials [57]. New printing capabilities are continuously emerging, however they also lead to barriers in costs and availability. Time and resolution are also limiting. It is possible to print scaffolds with nano-scale features [118], however higher resolutions lead to longer construction times. The inconsistencies in features near the limits of a printer’s resolution lead to unanticipated design behaviors [3], therefore motivating the need to investigate specific materials, printers, and design configurations in addition to developing general theories and models.

Assessment is necessary to validate scaffold performance and requires a combination of experiments for measurement and models to predict behavior. Extensive mechanical experiments and modeling is necessary due to the unique behaviors of varied designs, and require particular advancement in considering scaffolds as metamaterials that potentially have hierarchical features leading to further complexity [89]. Nutrient consumption and distribution is difficult to assess and there is a need to adjust biological growth predictions on 3D printed structures with modified tissue growth speeds based on nutrients delivered to the tissue [37]. In addition to experiments validating existing biological growth models in vivo [13], there is a need to incorporate modeling aspects such as mineralization. These models will provide a more complete picture for scaffold supported bone formation and are currently limited by the lack of available data and stochasticity inherent in biological systems.

These challenges provide a number of directions to advance tissue scaffold design, with a particular emphasis on bridging disciplines and processes to develop fully integrated approaches that could extend to tissue engineering applications beyond bone. Advances in tissue scaffold development are also informed through advances in related fields, such as recent investigations in how material degradation of metal implants influences the local biology via corrosion [119]. Overcoming these and the many other challenges present has the potential to significantly improve personalized health and medical approaches, therefore leading to improved quality of life and longevity.

## 8. Conclusions

In this review, the integration of design, fabrication, and assessment processes for porous scaffold material design was investigated, with a focus on clinical applications for bone tissue engineering. Integrated design encourages multidisciplinary refinement of 3D printed scaffolds configured as lattices, through efficient iteration that includes experiments and modeling. Numerous studies have provided the foundations for integrated scaffold design and here we highlighted opportunities and challenges in the field. Challenges include the need for developing new metamaterials and hierarchical porous structures with biocompatibility, in addition to conducting experiments to confirm influences from diverse printing processes and materials on lattice structure performance. Future research that addresses these and the many other identified challenges for integrated tissue scaffold design may significantly improve clinical outcomes, and facilitate innovative solutions for diverse healthcare applications.
